# Supplementary material for: Spike frequency adaptation supports network computations on temporally dispersed information
Source: eLife. 2021 Jul 26;10:e65459. doi: 10.7554/eLife.65459 (PMC8313230; doi:10.7554/eLife.65459)
Supplement: Transparent reporting form [file elife-65459-transrepform.docx]

***eLife’s* transparent reporting form**

We encourage authors to provide detailed information *within their submission* to facilitate the interpretation and replication of experiments. Authors can upload supporting documentation to indicate the use of appropriate reporting guidelines for health-related research (see [EQUATOR Network](http://www.equator-network.org/%20)), life science research (see the [BioSharing Information Resource](https://biosharing.org/)), or the [ARRIVE guidelines](http://www.plosbiology.org/article/info:doi/10.1371/journal.pbio.1000412) for reporting work involving animal research. Where applicable, authors should refer to any relevant reporting standards documents in this form.

If you have any questions, please consult our Journal Policies and/or contact us: [editorial@elifesciences.org](mailto:editorial@elifesciences.org).

**Sample-size estimation**

- You should state whether an appropriate sample size was computed when the study was being designed
- You should state the statistical method of sample size computation and any required assumptions
- If no explicit power analysis was used, you should describe how you decided what sample (replicate) size (number) to use

Please outline where this information can be found within the submission (e.g., sections or figure legends), or explain why this information doesn’t apply to your submission:

We did not perform experiments with biological subjects. Our spiking data for the analysis was generated through a simulation. We did not have to estimate the sample size, because we could use all the test episodes available (50000). However, we had to make sure that the requirements of the analysis are met, such as, for example, independent data points or balanced data set (the same number of points per condition). Only after these steps, the sample size was calculated and accepted as good enough - 135 data points (repeated, independent measurements) per condition and per neuron.

The analysis of spiking data, with results presented in Figures 4D, 4E, 4F is described in detail in the section “Analysis of spiking data for Duplication/Reversal task” (the last section of Methods).

**Replicates**

- You should report how often each experiment was performed
- You should include a definition of biological versus technical replication
- The data obtained should be provided and sufficient information should be provided to indicate the number of independent biological and/or technical replicates
- If you encountered any outliers, you should describe how these were handled
- Criteria for exclusion/inclusion of data should be clearly stated
- High-throughput sequence data should be uploaded before submission, with a private link for reviewers provided (these are available from both GEO and ArrayExpress)

Please outline where this information can be found within the submission (e.g., sections or figure legends), or explain why this information doesn’t apply to your submission:

In our case, an experiment corresponds to a test episode, with the spiking activity recorded for all neurons simultaneously.

To analyze the selectivity of a neuron, we used multiple data points per condition, i.e., “measurements” were repeated per neuron, hence this would correspond to technical replication.

The results are presented in Figures 4D, 4E, 4F, and described in detail in the section “Analysis of spiking data for Duplication/Reversal task” (the last section of Methods).

**Statistical reporting**

- Statistical analysis methods should be described and justified
- Raw data should be presented in figures whenever informative to do so (typically when N per group is less than 10)
- For each experiment, you should identify the statistical tests used, exact values of N, definitions of center, methods of multiple test correction, and dispersion and precision measures (e.g., mean, median, SD, SEM, confidence intervals; and, for the major substantive results, a measure of effect size (e.g., Pearson's r, Cohen's d)
- Report exact p-values wherever possible alongside the summary statistics and 95% confidence intervals. These should be reported for all key questions and not only when the p-value is less than 0.05.

Please outline where this information can be found within the submission (e.g., sections or figure legends), or explain why this information doesn’t apply to your submission:

We conducted 3-way ANOVA, for the results presented in Figure 4D, and Welch’s t-test for the results shown in Figures 4E, 4F.

The analysis is described in detail in the section “Analysis of spiking data for Duplication/Reversal task” (the last section of Methods).

Since our p-values were very small, i.e., the order of the magnitude was about 10^(-6), we do not report them as exact values. However, tables with results after performing 3-way ANOVA, for each neuron are given as an Additional file (“source data”), relevant for Figures 4D, 4E, 4F.

(For large datasets, or papers with a very large number of statistical tests, you may upload a single table file with tests, Ns, etc., with reference to sections in the manuscript.)

**Group allocation**

- Indicate how samples were allocated into experimental groups (in the case of clinical studies, please specify allocation to treatment method); if randomization was used, please also state if restricted randomization was applied
- Indicate if masking was used during group allocation, data collection and/or data analysis

Please outline where this information can be found within the submission (e.g., sections or figure legends), or explain why this information doesn’t apply to your submission:

This does not completely apply to our case, because we did not perform experiments with biological subjects.

Instead of allocating samples into experimental groups, we had to choose randomly data points from the available test episodes, in order to make sure that the data points were independent of each other, and that the data set was balanced.

This is described in the section “Analysis of spiking data for Duplication/Reversal task” (the last section of Methods)

**Additional data files (“source data”)**

- We encourage you to upload relevant additional data files, such as numerical data that are represented as a graph in a figure, or as a summary table
- Where provided, these should be in the most useful format, and they can be uploaded as “Source data” files linked to a main figure or table
- Include model definition files including the full list of parameters used
- Include code used for data analysis (e.g., R, MatLab)
- Avoid stating that data files are “available upon request”

Please indicate the figures or tables for which source data files have been provided:

We plan to publish the code, together with the notebooks used for the analysis and the data files needed for such an analysis.
This will be available in the near future on our GitHub repository: <https://github.com/IGITUGraz>.
